# Maior Rigidez Arterial Prediz Doença Renal Crônica no Estudo de Coorte ELSA-Brasil

**DOI:** 10.36660/abc.20230409

**Published:** 2024-01-19

**Authors:** Júlia Cândido, Lidyane do Valle Camelo, Luisa Brant, Roberto Sá Cunha, José Geraldo Mill, Sandhi Maria Barreto

**Affiliations:** 1 Universidade Federal de Minas Gerais Belo Horizonte MG Brasil Universidade Federal de Minas Gerais, Belo Horizonte, MG – Brasil; 2 Universidade Federal do Espirito Santo Vitoria ES Brasil Universidade Federal do Espirito Santo, Vitoria, ES – Brasil

**Keywords:** Insuficiência Renal Crônica, Rigidez Arterial, Taxa de Filtração Glomerular

## Abstract

**Fundamento:**

A rigidez arterial pode afetar diretamente os rins, que são perfundidos passivamente por alto fluxo. No entanto, determinar se a relação entre rigidez arterial e função renal depende das condições de diabetes e hipertensão é uma questão controversa.

**Objetivo:**

Investigar a relação entre a rigidez arterial, por velocidade da onda de pulso carotídea-femoral (VOPcf), e a incidência de doença renal crônica (DRC) em indivíduos e verificar se essa associação está presente em indivíduos sem hipertensão e diabetes.

**Métodos:**

Estudo longitudinal com 11.647 participantes do ELSA-Brasil acompanhados por quatro anos (2008/10-2012/14). A VOPcf basal foi agrupada por quartil, de acordo com pontos de corte específicos com relação a sexo. A presença de DRC foi verificada pela taxa de filtração glomerular (TFGe-CKD-EPI) < 60 ml/min/1,73 m^2^ e/ou relação albumina/creatinina ≥ 30 mg/g. Modelos de regressão logística foram executados para toda a coorte e uma subamostra livre de hipertensão e diabetes no início do estudo, após ajuste para idade, sexo, raça, escolaridade, tabagismo, relação colesterol/HDL, índice de massa corporal, diabetes, uso de anti-hipertensivos, pressão arterial sistólica, frequência cardíaca e doenças cardiovasculares. A significância estatística foi fixada em 5%.

**Resultados:**

A chance de DRC foi de 42% (IC de 95%: 1,05;1,92) maior entre indivíduos no quartil superior da VOPcf. Entre os participantes normotensos e não diabéticos, os indivíduos do 2º, 3º e 4º quartis da VOPcf apresentaram maiores chances de desenvolver DRC, quando comparados aos do quartil inferior, sendo a magnitude dessa associação maior para aqueles do quartil superior (OR: 1,81 IC de 95%: 1,14;2,86).

**Conclusão:**

A maior VOPcf aumentou as chances de DRC, e sugere que esse efeito é ainda maior em indivíduos sem diabetes e hipertensão.

## Graphical abstract

**Figura Central:**
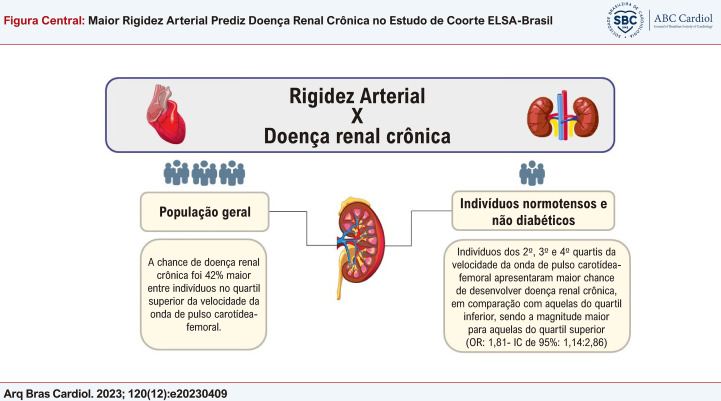
: Maior Rigidez Arterial Prediz Doença Renal Crônica no Estudo de Coorte ELSA-Brasil

## Introdução

A doença renal crônica (DRC) é um problema de saúde pública global com alta prevalência, morbidade e mortalidade.^1^ Em 2017, a DRC ocupou a 8ª posição como causa de morte no mundo,^2^ sendo também associada ao aumento do risco de eventos cardiovasculares.^3^ Em uma meta-análise de 110 estudos, a prevalência global estimada dos estágios 3 a 5 da DRC foi de 13%.^4^ Em 2013, a prevalência estimada de DRC em brasileiros foi de 6,7% com base na taxa de filtração glomerular estimada (TFGe).^5^ Na linha de base do estudo ELSA-Brasil, a prevalência de DRC em adultos, de 35 a 74 anos, foi de 8,9%.^6^ À medida que o número de idosos aumenta em todo o mundo, espera-se que a prevalência da DRC também aumente, especialmente em países de baixa e média renda.

A DRC está associada à disfunção vascular em diversos sítios anatômicos.^7^ Acredita-se que o aumento da rigidez arterial esteja associado à incidência e progressão da DRC e à mortalidade cardiovascular.^8,9^ A relação entre rigidez arterial e progressão da doença renal tem sido relatada em pacientes com DRC em estágio inicial^10,11^ e avançado,^8,12^ bem como na população geral.^13,14^ Contudo, alguns estudos detectaram associações fracas ou nenhuma associação.^15,16^

A maioria dos estudos anteriores abordou a relação entre rigidez arterial e TFGe ou DRC estabelecida. Existem poucos estudos longitudinais que investigam associações entre rigidez arterial e disfunção renal medida de acordo com a albuminúria ou a relação albumina/creatinina (RAC),^17^ e nenhum avaliou essa relação especificamente em indivíduos sem diabetes e hipertensão. No entanto, a RAC elevada é um marcador precoce de dano glomerular, particularmente em indivíduos com diabetes, hipertensão ou doença cardiovascular (DCV), sendo associada a maior mortalidade, independentemente da TFGe.^18,19^ Quanto à associação entre rigidez arterial e função renal, faz-se relevante saber se ela depende da diabetes ou da hipertensão.^13^ Essas condições de saúde interferem nas propriedades estruturais arteriais e podem explicar parte das associações entre aumento da rigidez arterial e a DRC.^20,21^ A rigidez arterial pode, aliás, preceder a elevação da pressão arterial e a DM.^22-24^

Este estudo tem como objetivo investigar as associações entre rigidez arterial e incidência de DRC, avaliada de acordo com os níveis de TFGe ou RAC, em cerca de quatro anos de acompanhamento. Além disso, o estudo investigou se tais associações são mantidas para indivíduos normotensos e não diabéticos, dois importantes fatores de risco para a DRC.

## Métodos

O ELSA-Brasil é um estudo multicêntrico prospectivo que envolveu 15.105 servidores públicos com idades entre 35 e 74 anos, recrutados em instituições de ensino superior e pesquisa de seis capitais brasileiras: São Paulo, Belo Horizonte, Porto Alegre, Rio de Janeiro, Salvador e Vitória.

Os dados foram coletados em dois momentos: visita 1 (2008 a 2010) e visita 2 (2012 a 2014). Em ambas as ocasiões, os participantes foram submetidos a entrevistas presenciais, avaliações clínicas, medidas antropométricas e exames laboratoriais e de imagem, realizados por auxiliares de pesquisa treinados e certificados.

O ELSA-Brasil foi aprovado pelos Comitês de Ética das instituições participantes. Todos os participantes assinaram um termo de consentimento livre e esclarecido antes da coleta de dados em ambas as visitas.

### População do estudo

**Figura 1:**
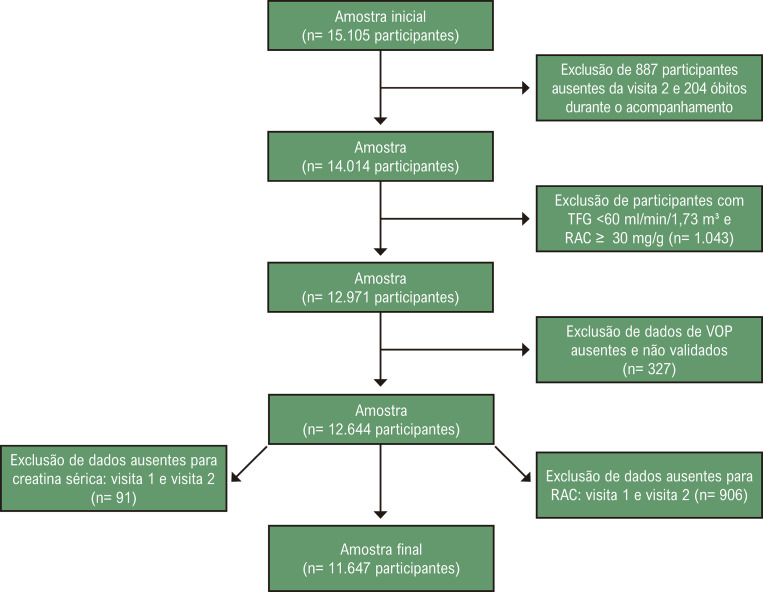
– Fluxograma de critérios de exclusão.

Dos 15.105 participantes que compareceram à primeira visita, 204 (1,4%) morreram durante o acompanhamento e 887 (5,9%) não compareceram à segunda visita. Dos 14.014 participantes que compareceram à segunda visita, aqueles livres de DRC na primeira visita foram elegíveis para participar do estudo (n=12.971). Indivíduos com VOP ausente ou não validada (n=327) e dados ausentes para creatinina sérica (n=91) ou RAC (n=906) em qualquer visita do estudo, também foram excluídos, resultando em uma amostra analítica de 11.647 participantes (Figura 1).

### Variáveis de Estudo

#### Doença renal crônica

A incidência de DRC na segunda visita de acompanhamento do ELSA-Brasil foi utilizada como variável de resposta neste estudo. A DRC (sim/não) foi definida como TFGe baixa (não/sim) e/ou RAC elevada (não/sim) na segunda visita, definida como TFGe <60 ml/min/1,73 m^2^ ou RAC ≥ 30 mg/g. Amostras de urina foram coletadas 12 horas antes das visitas. Amostras de sangue foram coletadas após jejum de 12 horas. Os níveis séricos de creatinina foram medidos pelo método colorimétrico enzimático de Jaffe (Advia 1200 Siemens, EUA). Os níveis urinários de creatinina e albumina foram medidos pelo método cinético de Jaffe (Advia 1200 Siemens, EUA) e por um ensaio imunoquímico (BN IINephelometer Siemens Dade Behring, EUA), respectivamente.

A TFGe foi calculada utilizando a equação *Chronic Kidney Disease Epidemiology Collaboration (CKD-EPI)* sem ajuste para raça/cor da pele.^6^

#### Rigidez arterial

A rigidez arterial foi medida como velocidade da onda de pulso carotídeo-femoral (VOPcf), determinada por meio de aparelho automático validado (Complior, Artech Medicale, França), com o paciente deitado em sala com temperatura controlada (20 ºC a 24 ºC). A VOPcf mede a rigidez da aorta, o território de interesse considerando seu papel principal no amortecimento do fluxo pulsátil, e o fato de ser um preditor independente de eventos cardiovasculares em diferentes populações. Antes da medição da VOPcf, a pressão arterial foi medida no braço direito com os pacientes deitados, utilizando um aparelho oscilométrico (Omron HRM 705 CP). A distância entre a fúrcula supraesternal e o pulso femoral direito foi medida com fita métrica. A circunferência abdominal não foi considerada. Sensores de pulso foram colocados nas artérias femoral e carótida direita, e as ondas de pulso foram visualizadas na tela do computador.^25^ A VOPcf foi calculada dividindo-se a distância entre a fúrcula supraesternal e o pulso femoral pelo atraso entre o pulso carotídeo e o pulso femoral,^25^ sendo a média aritmética de dez ciclos cardíacos consecutivos em ritmo cardíaco regular. Neste estudo, a distribuição da VOPcf variou por sexo e, portanto, os dados da VOPcf foram divididos em quartis específicos para sexo, correspondendo aos seguintes intervalos: <7,8; 7,8-8,6; 8,7-9,6; e >9,6 m/s, em mulheres; e <8,4; 8,4-9,2; 9,3-10,3; e >10,3 m/s, em homens. O 1º quartil foi utilizado como referência. Os mesmos pontos de corte do quartil de VOPcf foram usados para analisar as associações entre VOPcf e DRC em participantes não diabéticos e normotensos.

## Covariáveis

As covariáveis foram obtidas no início do estudo. As variáveis sociodemográficas incluíram idade, sexo, raça/cor autorreferida (preta, branca, parda, outras) e escolaridade (superior, médio, fundamental completo ou fundamental incompleto). As variáveis comportamentais incluem tabagismo e índice de massa corporal (IMC). As variáveis clínicas foram relação entre colesterol total e lipoproteína de alta densidade (HDL), diabetes, DCV, pressão arterial sistólica (PAS), frequência cardíaca (FC) e uso de anti-hipertensivos.

O IMC foi obtido pelo peso corporal em quilogramas (kg) dividido pela altura em metros quadrados (m^2^), conforme técnicas padronizadas.^26^ O tabagismo foi considerado como (sim) ou (não). A atividade física foi determinada pelo domínio AFL do Questionário Internacional de Atividade Física (IPAQ). Esse instrumento foi validado na população brasileira e inclui questões referentes à frequência, duração e intensidade de atividades com duração de dez ou mais minutos.^27^ O colesterol total e HDL foram medidos em amostras de sangue obtidas após jejum de 12 horas, utilizando métodos colorimétricos enzimáticos padronizados. O diabetes foi definido por meio de confirmação por diagnóstico médico e/ou uso de medicação antidiabética e/ou glicemia em jejum ≥126 mg/dL e/ou teste oral de tolerância à glicose de 75 g ≥200 mg/dL e/ou HbA1c≥6,5%. A PAS foi definida como ≥140 mmHg, medida pelo método oscilométrico por meio de dispositivo (Omron HEM 705CPINT) no braço direito após repouso de cinco minutos na posição sentada em uma sala silenciosa com temperatura controlada (20 – 24 °C). Três medições foram realizadas em intervalos de um minuto, e as médias das duas últimas medidas foram utilizadas.^25^ A hipertensão foi definida como PAS ≥140 mmHg, pressão arterial diastólica (PAD) ≥ 90 mmHg ou uso de medicamentos anti-hipertensivos. O uso de anti-hipertensivos foi autorreferido e/ou determinado por meio de exame de blister, embalagens e receitas médicas. As DCVs foram autorreferidas (sim/não), incluindo diagnósticos de infarto agudo do miocárdio, cirurgia de revascularização cardíaca, insuficiência cardíaca e acidente vascular. A FC foi medida três vezes após repouso de cinco minutos, com os participantes na posição sentada, utilizando um aparelho oscilométrico validado (Omron HEM-705 CP).

Na análise, a amostra foi estratificada de acordo com a condição de hipertensão e diabetes no início do estudo.

## Análise de dados

As características basais da população geral do estudo e da subamostra sem diabetes e hipertensão foram descritas como proporções e médias. As variáveis categóricas foram descritas como proporções e as variáveis contínuas como médias e desvios padrão.

Modelos de regressão logística investigaram as associações entre os quartis basais da VOPcf e a incidência de DRC de acordo com a TFGe ou RAC na visita 2. Após o modelo bruto, os seguintes fatores de confusão foram adicionados à análise de toda a amostra. No modelo 1, foram acrescentados idade, sexo, raça/cor e escolaridade. No modelo 2, foram incluídos tabagismo, atividade física, IMC e relação colesterol total-HDL. Por fim, o uso de anti-hipertensivos, a PAS, o diabetes, a FC e as DCVs foram acrescentados no modelo final. A mesma estratégia analítica foi repetida com participantes que não apresentavam hipertensão ou diabetes no início do estudo. Assim, diabetes e uso de medicação anti-hipertensiva não foram incluídos nos modelos finais desta análise.

A normalidade dos dados foi testada graficamente, por meio de histogramas. O nível de significância estatística foi fixado em 5%. As análises foram realizadas por meio de software (Stata 14.0, Stata Corporation, College Station, Estados Unidos).

## Resultados

Os participantes da amostra geral tinham idade de 51±8 anos. A maioria dos participantes era do sexo feminino (54,8%), raça/cor da pele autodeclarada como branca (53,3%), com ensino superior completo (54,2%). A média da VOPcf foi de 9,1±1,7 m/s (Tabela 1). O tempo médio de acompanhamento entre as visitas foi de 3,8±0,42 anos. A incidência global de DRC foi de 5,6%, definida pela alteração da TFGe ou RAC, que foram de 5,7% e 2,5%, respectivamente, enquanto a incidência de DRC em participantes sem diabetes ou sem hipertensão foi de 3,2% (TFGe baixa foi de 2% e RAC alta foi de 1,3%). A incidência de DRC na amostra geral foi maior em homens, pretos, com baixa escolaridade e em participantes com diabetes e hipertensão (Tabela 2).

**Tabela 1:** – Características descritivas dos participantes da linha de base do Estudo Longitudinal de Saúde do Adulto Brasileiro (ELSA-Brasil), 2008-2010, (N=11.647)

Características	% ou média (DP)	
%, média (DP)	População geral	Participantes normotensos e não diabéticos
	N: 11.647	N: 7.309
Idade (anos), média (DP)	51 (8)	49 (8)
Sexo (%)		
Feminino	54,8	58
Raça/cor, (%)		
Branca	53,3	57
Parda	27,7	27,3
Preta	15,4	12,2
Outros	3,5	3,4
Nível de escolaridade, (%)		
Ensino superior	54,9	59,5
Ensino médio completo	34,6	32,6
Ensino fundamental	5,9	4,8
Ensino médio incompleto	4,5	2,9
Diabetes mellitus, (%)	12,7	-
Hipertensão, (%)	30,3	-
Uso de anti-hipertensivo, (%)	24,2	-
Frequência cardíaca (bpm), média (DP)	70 (10)	69 (9)
Pressão arterial sistólica (mmHg), média (DP)	120 (16)	113 (11)
Pressão arterial diastólica (mmHg), média (DP)	76 (10)	72 (8)
VOPcf (m/s) média (DP)	9,1 (1.6)	8,6 (1.3)

**Tabela 2:** – Incidência cumulativa de doença renal crônica após aproximadamente quatro anos (2008/2010–2012/2014), de acordo com características dos participantes da linha de base em toda a amostra e na subamostra de participantes normotensos e não diabéticos

Características	Incidência (%)	
	População geral	Participantes normotensos e não diabéticos
Total	5,6	3,2
Idade (anos)		
34-44	2,0	1,3
45-54	4,0	2,9
55-64	7,4	5,8
65-75	19,6	13,3
Sexo		
Feminino	5,2	2,7
Masculino	5,3	3,5
Raça/cor, (%)		
Branca	5,0	3,0
Parda	4,6	3,2
Preta	7,1	4,2
Outros	4,3	2,8
Nível de escolaridade, (%)		
Ensino superior	4,6	2,9
Ensino médio completo	5,3	3,1
Ensino fundamental	7,8	4,9
Ensino médio incompleto	7,5	5,6
Diabetes mellitus		-
Sim	12,7	
Não	4,5	
Hipertensão		-
Sim	9,5	
Não	3,7	
Uso de anti-hipertensivo		-
Sim	10	
Não	4,1	

**Figura 2:**
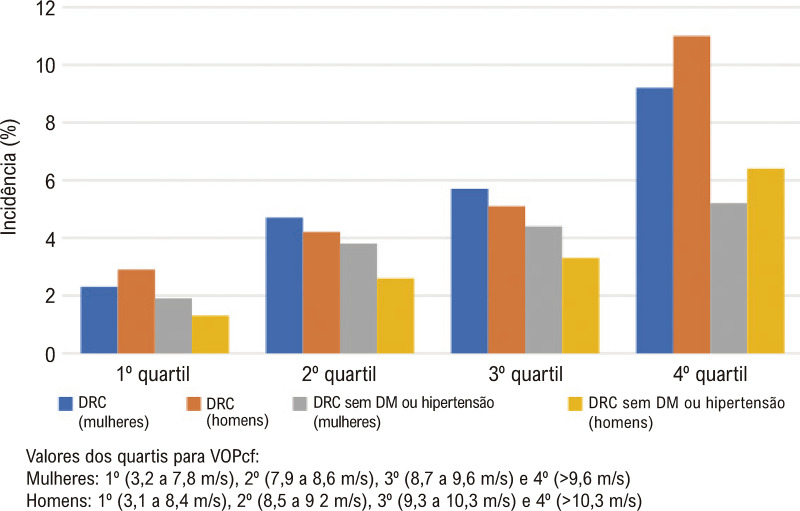
– Incidência cumulativa de doença renal crônica (DRC) após aproximadamente quatro anos de acompanhamento (2008/2010–2012/2014), segundo quartis de velocidade da onda de pulso específicos para sexo em toda a amostra e na subamostra sem diabetes mellitus e sem hipertensão.

Como podemos observar na Figura 2, quanto maior o quartil da VOP, maior a incidência de DRC, em ambos os sexos. Um padrão semelhante foi observado em participantes normotensos não diabéticos, mas neste grupo as diferenças na incidência de DRC, de acordo com os quartis da VOP, parecem menos pronunciadas. Em ambas as populações, ela foi mais pronunciada nos homens do 4º quartil (Figura 2).

No geral, quanto maior a VOP, maiores as chances de DRC ao longo de quatro anos de acompanhamento na população geral (Tabela 3). Após ajuste para variáveis sociodemográficas, esse padrão permaneceu, mas a magnitude das associações diminuiu substancialmente. No modelo final, observamos que apenas o 4º quartil permaneceu estatisticamente significativo, mostrando que homens com VOP superior a 10,3 m/s e mulheres com VOP superior a >9,6 m/s apresentaram 42% (IC de 95%: 1,05;1,92) mais chances de DRC após quatro anos de acompanhamento. A análise dos participantes normotensos e não diabéticos produziu resultados semelhantes, mas com maiores magnitudes de associações (Tabela 4). No modelo final, incluindo apenas participantes normotensos e não diabéticos, houve um claro gradiente dose-resposta na associação entre o quartil da VOPcf e as chances de DRC, atingindo um OR de 1,81 (IC de 95%: 1,14;2,86) entre os indivíduos do quartil superior em comparação com os do quartil inferior (Tabela 4).

**Tabela 3:** – Associação entre quartis de velocidade da onda de pulso e incidência de doença renal crônica em toda a amostra, após quatro anos de acompanhamento (2008/2010–2012/2014)

Modelos	Doença renal crônica (N:11.647) OR (IC de 95%)
Modelo Univariável	
1º quartil	Ref.
2º quartil	1,74 (1,31;2,31)***
3º quartil	2,16 (1,64;2,83)***
4º quartil	4,14 (3,22;5,33)***
Modelo 1: modelo 0 + idade, sexo, raça/cor e escolaridade	
1º quartil	Ref.
2º quartil	1,46 (1,09;1,94)*
3º quartil	1,50 (1,13; 1,99)
4º quartil	1,94 (1,47;2,55)***
Modelo 2: modelo 1 + tabagismo, atividade física, colesterol total/HDL-C e IMC	
1º quartil	Ref.
2º quartil	1,39 (1,04;1,86)*
3º quartil	1,42 (1,06;1,88)*
4º quartil	1,79 (1,36; 2,37)***
Modelo final: modelo 3 + DM, uso de anti-hipertensivo, PAS, FC e DCV	
1º quartil	Ref.
2º quartil	1,32 (0,98; 1,78)
3º quartil	1,31 (0,98; 1,76)
4º quartil	1,42 (1,05;1,92)*

OR: odds ratio obtida por regressão logística múltipla. IC, intervalo de confiança. IMC: índice de massa corporal FC: frequência cardíaca. PAS: pressão arterial sistólica. DCV: doença cardiovascular. *p<0,05 **p<0,01 ***p<0,001

**Tabela 4:** – Associação entre quartis de velocidade da onda de pulso e incidência de doença renal crônica na subamostra de participantes normotensos e não diabéticos, após quatro anos de acompanhamento (2008/2010–2012/2014)

Modelos	Doença renal crônica (N:7.189) OR (IC de 95%)
Modelo Univariável	
1º quartil	Ref.
2º quartil	1,99 (1,35;2,94)***
3º quartil	2,39 (1,62;3,62)***
4º quartil	3,49 (2,35;5,20)***
Modelo 1: modelo 0 + idade, sexo, raça/cor e escolaridade	
1º quartil	Ref.
2º quartil	1,66 (1,12;2,46)*
3º quartil	1,65 (1,17;2,47)*
4º quartil	1,79 (1,17; 2,74)
Modelo 2: modelo 1 + tabagismo, atividade física, colesterol total/HDL-C e IMC	
1º quartil	Ref.
2º quartil	1,58 (1,06;2,35)*
3º quartil	1,61 (1,07;2,41)*
4º quartil	1,76 (1,14; 2,70)
Modelo final: modelo 3 + PAS, FC e DCV	
1º quartil	Ref.
2º quartil	1,61 (1,08;2,41)*
3º quartil	1,63 (1,07;2,47)*
4º quartil	1,81 (1,14;2,86)*

OR: odds ratio obtida por regressão logística múltipla. IC, intervalo de confiança. IMC: índice de massa corporal FC: frequência cardíaca. PAS: pressão arterial sistólica. DCV: doença cardiovascular *p<0,05 **p<0,01 ***p<0,001

## Discussão

Nesta grande coorte multicêntrica brasileira de adultos, as chances de desenvolver DRC em quatro anos de acompanhamento, com base na TFGe e/ou RAC, foram 42% maiores em indivíduos no quartil superior em relação aos do quartil inferior, após ajustes para características sociodemográficas, comportamentais e clínicas. Quando apenas indivíduos normotensos e não diabéticos basais foram contabilizados, as magnitudes das associações dos quartis da VOPcf e DRC, em comparação com o quartil inferior, foram maiores do que aquelas observadas na amostra geral e, também, progressivas - quase dobrando no quartil superior.

Estudos sugerem que a VOPcf >10 m/s aumenta o risco cardiovascular, sendo um importante marcador de risco clínico.^8,28^ Neste estudo, o 4º quartil da VOPcf corresponde a valores de VOPcf acima de 10,3 m/s em homens e acima de > 9,6 m/s em mulheres. Uma metanálise global de 167 estudos, totalizando 509.743 participantes, apresentou informações significativas sobre as diferenças entre os sexos nas medidas da VOPcf, nas quais os homens apresentavam maior rigidez arterial do que as mulheres, principalmente na idade adulta jovem e até 60 anos, justificando o uso dos dados da VOPcf divididos em quartis específicos para sexo.^29^

Os achados deste estudo corroboram outros estudos longitudinais que investigaram as relações entre rigidez arterial e incidência de disfunção renal, definida de acordo com a TFGe na população geral^24,26,11^ e em indivíduos com DRC^7,10^ ou comorbidades, como DM^30^ e hipertensão.^12^ Itano et al. (2020) também descobriram que indivíduos no quartil superior de rigidez arterial apresentaram aumento na incidência de DRC ao longo de um seguimento médio de 3,1 anos, quando comparados com os demais quartis agrupados como referência. Porém, diferentemente de nós, utilizaram o índice vascular cardio-tornozelo e o quartil mais alto correspondeu a >8,1m/s.^26^ Townsend (2018) acompanhou 2.795 participantes, com idade média de 60 anos, por 4,9±2,1 anos e descobriu que indivíduos no tercil superior da VOPcf (>10,3 m/s) tinham 37% mais risco de desenvolver DRC (IC de 95%: 1,05-1,80), bem como um risco 25% maior de ter doença renal em estágio terminal ou a TFGe reduzida pela metade (RR: 1,25; 0,98-1,58).^7^

Os resultados deste estudo trazem contribuições significativas para os poucos estudos longitudinais de base populacional que utilizaram a RAC como marcador da função renal. Os achados de uma coorte chinesa^31^ de 7.154 indivíduos, com idade média de 54 anos, mostraram uma associação linear da rigidez arterial com o risco de DRC, em que cada aumento de 1 m/s na VOP foi associado a uma chance 15% maior de proteinúria (IC de 95%: 1,07-1,23) após acompanhamento de três anos. No entanto, estudos de coortes menores não conseguiram revelar associações significativas entre os valores da VOPcf e a incidência de microalbuminúria em modelos totalmente ajustados para fatores de risco cardiovascular. No estudo Framingham Offspring, uma VOPcf mais alta foi modestamente associada à microalbuminúria em 568 participantes com RAC <30 mg/g na linha de base, após ajuste para idade e sexo, mas não no modelo final ajustado para todos os fatores de risco após um período de sete a dez anos de acompanhamento.^15^ Associações significativas entre VOPcf e RAC são mais comuns em estudos transversais.^32^

Em indivíduos normotensos e não diabéticos, a magnitude dessas associações foi ligeiramente maior, sugerindo que o impacto de uma maior VOPcf no risco de DRC é mais pronunciado em indivíduos previamente saudáveis. Esta descoberta pode refletir que: 1) a maior rigidez arterial está associada *per se* à incidência de DRC, e não como consequência de hipertensão e diabetes, ou 2) confusão residual de hipertensão e diabetes nos modelos aplicados à população geral. Relações significativas entre VOPcf e função renal foram demonstradas em indivíduos normotensos com insuficiência renal leve a moderada.^33^ Em uma análise transversal, a rigidez arterial foi associada a maiores chances de DRC e disfunção renal em indivíduos sem hipertensão e diabetes, sugerindo que as relações entre rigidez arterial e disfunção renal não são inteiramente explicadas por tais condições.^34^ Considerando que a maior rigidez arterial aumenta o risco de hipertensão e diabetes,^23,24^ é possível levantar a hipótese de que ela também poderia estar diretamente implicada na gênese da DRC, independentemente da hipertensão e do diabetes. Assim, sugere-se que o efeito do aumento da rigidez arterial na incidência de DRC é independente da hipertensão e/ou diabetes, os principais fatores de risco para disfunção renal.^12^ E a menor magnitude da associação na população geral pode ser justificada por uma possível confusão residual por esses fatores.

Alguns mecanismos podem explicar associações entre aumento da rigidez arterial e maior incidência de DRC. Devido à sua baixa impedância vascular, a circulação renal é sensível às oscilações da pressão arterial e ao aumento da pulsatilidade, resultantes do aumento da rigidez arterial.^35^ A maior rigidez da camada média em grandes artérias pode afetar a capacidade dos vasos renais de atenuar as alterações da pressão arterial a cada ejeção sistólica. Assim, à medida que a aorta se torna mais rígida, o estresse pulsátil dos vasos sanguíneos periféricos aumenta, levando a danos microvasculares, hiperfiltração e esclerose e hipertrofia glomerular, o que resulta em diminuição da área de superfície de filtração e menor TFG.^35,36^ Por sua vez, o estresse hemodinâmico nos vasos renais pode levar à disfunção endotelial e à isquemia microvascular, que interferem na permeabilidade da barreira glomerular^12,31^ e permitem maior excreção urinária de albumina.^37^ Esses mecanismos podem ocorrer mesmo em indivíduos livres de hipertensão e diabetes.

Os pontos fortes deste estudo incluem o grande tamanho da coorte, a coleta de dados abrangente e rigorosa e a alta taxa de retenção (92,7%), a determinação da DRC de acordo com a TFG ou RAC e a análise de um subconjunto de indivíduos normotensos e não diabéticos. No entanto, as relações entre rigidez arterial e DRC podem ser examinadas mais detalhadamente com um acompanhamento mais longo, dada a lenta progressão da disfunção renal,^38^ particularmente da albuminúria. Além disso, a TFGe e a RAC foram estimadas em um único ponto no tempo. Embora esta seja uma prática comum em grandes estudos epidemiológicos, como o ELSA-Brasil, a definição de DRC de acordo com alterações renais que persistem por três meses ou mais^38^ não foi considerada. A variabilidade intraindividual na excreção urinária de albumina e creatinina também foi relatada.^39^

## Conclusão

A maior rigidez arterial aumenta o risco de DRC na população geral e em indivíduos normotensos e não-diabéticos, sugerindo que a associação encontrada não é dependente dessas comorbidades. Dado que a doença renal crônica está associada a um maior risco de morte, eventos cardiovasculares e morbidade, esses resultados destacam a relevância da saúde vascular na prevenção do desenvolvimento da DRC.
