# Supplementary material for: Maior Rigidez Arterial Prediz Doença Renal Crônica no Estudo de Coorte ELSA-Brasil
Source: Arq Bras Cardiol. 2024 Jan 19;120(12):e20230409. [Article in Portuguese] doi: 10.36660/abc.20230409 (PMC11021122; doi:10.36660/abc.20230409)
Supplement: Translation

# Translation: Higher Arterial Stiffness Predicts Chronic Kidney Disease in Adults: The ELSA-Brasil Cohort Study

**Authors:** Júlia Cândido, Lidyane do Valle Camelo, Luisa Brant, Roberto Sá Cunha, José Geraldo Mill, Sandhi Maria Barreto

**Affiliations:** 1 Brazil Universidade Federal de Minas Gerais, Belo Horizonte, MG – Brazil; 2 Brazil Universidade Federal do Espirito Santo, Vitoria, ES – Brazil

**Keywords:** Chronic Kidney Disease, Arterial Stiffness, Glomerular Filtration Rate

## Abstract

**Background:**

Arterial stiffening can directly affect the kidneys, which are passively perfused by a high flow. However, whether the relation between arterial stiffness and renal function depends on diabetes and hypertension conditions, is a matter of debate.

**Objective:**

To investigate the relationship between arterial stiffening by carotid-to-femoral pulse wave velocity (cfPWV) and chronic kidney disease (CKD) incidence in individuals and verify whether this association is present in individuals without hypertension and diabetes.

**Methods:**

A longitudinal study of 11,647 participants of the ELSA-Brasil followed up for four years (2008/10-2012/14). Baseline cfPWV was grouped per quartile, according to sex-specific cut-offs. Presence of CKD was ascertained by glomerular filtration rate (eGFR-CKD-EPI) < 60 ml/min/1.73 m^2^ and/or albumin-to-creatinine ratio ≥ 30 mg/g. Logistic regression models were run for the whole cohort and a subsample free from hypertension and diabetes at baseline, after adjustment for age, sex, race, schooling, smoking, cholesterol/HDL ratio, body mass index, diabetes, use of antihypertensive, systolic blood pressure, heart rate, and cardiovascular disease. Statistical significance was set at 5%.

**Results:**

The chance of CKD was 42% (CI 95%: 1.05;1.92) greater among individuals in the upper quartile of cfPWV. Among normotensive, non-diabetic participants, individuals in the 2^nd^, 3^rd^, and 4^th^ quartiles of cfPWV presented greater chances of developing CKD, as compared to those in the lower quartile, and the magnitude of this association was the greatest for those in the upper quartile (OR: 1.81 CI 95%: 1.14;2.86).

**Conclusion:**

Higher cfPWV increased the chances of CKD and suggests that this effect is even greater in individuals without diabetes and hypertension.

## Introduction

Chronic kidney disease (CKD) is a global public health problem due to its high prevalence, morbidity, and mortality.^1^ In 2017, CKD ranked 8^th^ as a cause of death worldwide,^2^ and is also associated with an increased risk of cardiovascular events.^3^ In a meta-analysis of 110 studies, the estimated global prevalence of stage 3 to 5 CKD was 13%.^4^ In 2013, the estimated prevalence of CKD in Brazilians was 6.7% based on the estimated glomerular filtration rate (eGFR).^5^ In the baseline of the ELSA-Brasil study, the prevalence of CKD in adults, aged 35 to 74, was 8.9%.^6^ As the number of older persons grows worldwide, the prevalence of CKD is expected to rise, particularly in low- and middle-income countries.

CKD is associated with vascular dysfunction in several anatomical sites.^7^ Increased arterial stiffness is thought to be associated with the incidence and progression of CKD and with cardiovascular mortality.^8,9^ The relation between arterial stiffness and kidney disease progression has been reported in patients at early^10,11^and advanced CKD,^8,12^ as well as in the general population.^13,14^ However, some studies failed to detect any association or detected weak ones.^15,16^

Most previous studies have addressed the relation between arterial stiffness and eGFR or established CKD. Longitudinal studies investigating associations between arterial stiffness and kidney dysfunction measured according to albuminuria- or albumin-to-creatinine ratio (ACR) are scarce,^17^ and none have evaluated this relation specifically in individuals without diabetes and hypertension. However, raised ACR levels are early markers of glomerular damage, especially in individuals with diabetes, hypertension, or cardiovascular disease (CVD), and are associated with higher mortality regardless of eGFR.^18,19^ Regarding the association between arterial stiffness and kidney function, it is relevant to know if it depends on diabetes or hypertension.^13^ These health conditions interfere with arterial structural properties and may explain part of the associations between increased arterial stiffness and CKD.^20,21^ Indeed, arterial stiffness may precede blood pressure elevation and diabetes.^22-24^

This study aims to investigate the associations between arterial stiffness and CKD incidence, assessed according to eGFR or ACR levels, in about four years of follow-up. Moreover, it investigated whether these associations are maintained for normotensive and non-diabetic individuals, two major risk factors for CKD.

## Methods

ELSA-Brasil is a prospective multicenter study involving 15,105 civil servants aged 35 to 74, recruited from higher education and research institutions in six Brazilian capital cities: São Paulo, Belo Horizonte, Porto Alegre, Rio de Janeiro, Salvador, and Vitória.

Data was collected on two occasions: visit 1 (2008 to 2010) and visit 2 (2012 to 2014). On both occasions, participants were submitted to face-to-face interviews, clinical assessments, anthropometric measurements, and laboratory and imaging tests conducted by trained and certified research assistants.

ELSA-Brasil was approved by the Ethics Committees of participating institutions. All participants signed an informed consent term before data collection on both visits.

### Study population

Of the 15,105 participants who attended the first visit, 204 (1.4%) died during the follow-up and 887 (5.9%) did not attend the second visit. Of the 14,014 participants who attended the second visit, those free of CKD at the first visit were eligible to participate (n=12,971). This study also excluded individuals with missing or non-validated PWV (n=327) and missing data of serum creatinine data (n=91) or ACR data (n=906) at any study visit, resulting in an analytical sample of 11,647 participants (Figure 1).

**Figure 1:** – Exclusion criteria flowchart.

### Study variables

#### Chronic kidney disease

CKD incidence in the second ELSA-Brasil follow-up visit was used as a response variable in this study. CKD (yes/no) was defined as low eGFR (no/yes) and/or high ACR (no/yes) in the second visit, defined as eGFR <60 ml/min/1.73 m ^2^ or ACR ≥ 30 mg/g. Urine samples were self-collected 12 hours prior to visits. Blood samples were collected after a 12-hour fasting. Serum creatinine levels were measured using the enzymatic colorimetric Jaffe method (Advia 1200 Siemens, USA). Urine creatinine and albumin levels were measured using the kinetic Jaffe method (Advia 1200 Siemens, USA) and an immunochemical assay (BN IINephelometer Siemens Dade Behring, USA), respectively.

The eGFR was calculated using the Chronic Kidney Disease Epidemiology Collaboration (CKD-EPI) equation with no adjustment for race/skin color.^6^

#### Arterial stiffness

Arterial stiffness was measured as carotid-femoral pulse wave velocity (cfPWV) determined using a validated automatic device (Complior, Artech Medicale, France), with the patient lying down in a temperature-controlled room (20 ºC to 24 ºC). cfPWV measures the stiffness of the aorta, the territory of interest given its primary role in dampening the pulsatile flow, and the fact that it is an independent predictor of cardiovascular events in different populations. Prior to cfPWV measurement, blood pressure was measured at the right arm with patients lying down, using an oscillometric device (Omron HRM 705 CP). The distance from the suprasternal notch to the right femoral pulse was taken using a measuring tape. The abdominal circumference was not considered. Pulse sensors were placed on the right femoral and carotid arteries, and pulse waves were visualized on a computer screen.^25^ cfPWV was calculated by dividing the distance from the suprasternal notch to the femoral pulse by the delay between the carotid and the femoral pulse,^25^ being the arithmetic mean of ten consecutive cardiac cycles at regular heart rhythm. In this study, because cfPWV distribution varied by sex, cfPWV data was divided into sex-specific quartiles, corresponding to the following intervals: <7.8; 7.8-8.6; 8.7-9.6; and >9.6 m/s, in women; and <8.4; 8.4-9.2; 9.3-10.3; and >10.3 m/s, in men. The 1^st^ quartile was used as reference. The same cfPWV quartile cut-offs were used to analyze the associations between cfPWV and CKD in non-diabetic, normotensive participants.

## Covariates

The covariates were obtained at baseline. Sociodemographic variables included age, sex, self-reported race/color (black, white, brown, other), and level of education (higher, secondary, complete primary, or incomplete primary education). Behavioral variables comprised smoking and body mass index (BMI). The clinical variables were total- to high-density lipoprotein cholesterol (HDL) ratio, diabetes, CVD, systolic blood pressure (SBP), heart rate (HR), and use of antihypertensive drugs.

BMI was obtained by body weight in kilograms (kg) divided by height in meters square (m^2^ ), as per standardized techniques.^26^ Smoking was considered current (yes) or not (no). Physical activity was determined by the LTPA domain of the International Physical Activity Questionnaire (IPAQ). This instrument has been validated in the Brazilian population and includes questions regarding the frequency, duration, and intensity of activities lasting ten or more minutes.^27^ Total and HDL cholesterol were measured in blood samples obtained after 12 hours of fasting, using standardized enzymatic colorimetric methods. Diabetes was defined as a medical diagnosis of diabetes and/or use of antidiabetic medication and/or fasting glucose ≥126 mg/dL, and/or 75-g oral glucose tolerance test ≥200 mg/dL, and/or HbA1c≥6.5%. SBP was defined as ≥140 mmHg, measured by the oscillometric method (Omron HEM 705CPINT) device on the right arm after a five-minute rest in a sitting position in a quiet, temperature-controlled room (20 – 24 °C). Three measurements were taken at one-minute intervals, and the means of the last two measurements were used.^25^ Hypertension was defined as SBP ≥140 mmHg, diastolic blood pressure (DBP) ≥ 90 mmHg, or antihypertensive drug use. Antihypertensive drug use was self-reported or/and by examining blisters, packages, and prescriptions. CVD was self-reported (yes/no), including diagnoses of acute myocardial infarction, cardiac revascularization surgery, heart failure, and stroke. HR was measured three times after a five-minute rest with participants in the seated position, using a validated oscillometric device (Omron HEM-705 CP).

During analysis, the sample was stratified according to hypertension and diabetes condition at baseline.

## Data analysis

The baseline characteristics of the overall study population and of the subsample without diabetes and hypertension were described as proportions and means. Categorical variables were described as proportions, and continuous variables as means and standard deviations.

Logistic regression models investigated the associations between baseline cfPWV quartiles and the incidence of CKD according to eGFR or ACR in visit 2. After the crude model, the following confounders were added to the analysis of the whole sample. In model 1, age, sex, race/color, and schooling were added. In model 2, smoking, physical activity, BMI, and total cholesterol-HDL ratio were included. Finally, the use of antihypertensive drugs, SBP, diabetes, HR, and CVD were added to the final model. The same analytical strategy was repeated with participants who did not have hypertension or diabetes at baseline. Hence, diabetes and the use of antihypertensive medication were not included in the final models in this analysis.

Data normality was assessed graphically, using histograms. The level of statistical significance was set at 5%. Analyses were conducted using software (Stata 14.0, Stata Corporation, College Station, United States).

## Results

Participants in the general sample were aged 51±8 years. Most participants were female (54.8%), self-declared race/skin color as white (53.3%), and attained a higher education degree (54.2%). The mean cfPWV was 9.1±1.7 m/s (Table 1). Mean follow-up time between visits was 3.8±0.42 years. The overall incidence of CKD was 5.6%, defined by alteration of eGFR or ACR, which were 5.7% and 2.5%, respectively, whereas the incidence of CKD in participants without diabetes or without hypertension was 3.2% (low eGFR was 2% and high ACR was 1.3%). The incidence of CKD in the overall sample was higher in men, black people, people with low education, and participants with diabetes and hypertension (Table 2).

**Table 1:** – Descriptive characteristics of participants from baseline of the Brazilian Longitudinal Study of Adult Health (ELSA-Brasil), 2008-2010, (N=11,647)

Characteristics	% or mean (SD)	
%, mean (SD)	General population	Normotensive, non-diabetic participants
**N: 11,647**	**N: 7,309**
Age (years), mean (SD)	51 (8)	49 (8)
Sex, (%)		
Female	54.8	58
Race/color, (%)		
White	53.3	57
Brown	27.7	27.3
Black	15.4	12.2
Other	3.5	3.4
Education, (%)		
Undergraduate studies	54.9	59.5
High school graduate	34.6	32.6
Middle school	5.9	4.8
Middle school dropout	4.5	2.9
Diabetes mellitus, (%)	12.7	-
Hypertension, (%)	30.3	-
Use of antihypertensive, (%)	24.2	-
Heart rate (bpm), mean (SD)	70 (10)	69 (9)
Systolic blood pressure (mmHg), mean (SD)	120 (16)	113 (11)
Diastolic blood pressure (mmHg), mean (SD)	76 (10)	72 (8)
cfPWV (m/s) mean (SD)	9.1 (1.6)	8.6 (1.3)

**Table 2:** – Cumulative incidence of chronic kidney disease after approximately four years (2008/2010–2012/2014), according to characteristics of baseline participants in the entire sample and in the normotensive, non-diabetic participants subsample

Characteristics	Incidence (%)	
	General population	Normotensive, non-diabetic participants
Total	5.6	3.2
Age (years)		
34-44	2.0	1.3
45-54	4.0	2.9
55-64	7.4	5.8
65-75	19.6	13.3
Sex		
Female	5.2	2.7
Male	5.3	3.5
Race/color, (%)		
White	5.0	3.0
Brown	4.6	3.2
Black	7.1	4.2
Other	4.3	2.8
Education, (%)		
Undergraduate studies	4.6	2.9
High school graduate	5.3	3.1
Middle school	7.8	4.9
Middle school dropout	7.5	5.6
Diabetes mellitus		-
Yes	12.7	
No	4.5	
Hypertension		-
Yes	9.5	
No	3.7	
Use of antihypertensive		-
Yes	10	
No	4.1	

As we can see in Figure 2, the higher the PWV quartile, the higher the incidence of CKD, in both genders. A similar pattern was observed in non-diabetic normotensive participants, but in this group, the differences in the incidence of CKD according to PWV quartiles are less pronounced. In both populations, it was more pronounced in men in the 4^th^ quartile (Figure 2).

**Figure 2:** – Cumulative incidence of chronic kidney disease (CKD) after approximately four years of follow-up (2008/2010–2012/2014), according to sex-specific quartiles of pulse wave velocity in the entire sample and in the subsample without diabetes mellitus and hypertension.

Overall, the higher the PWV, the higher the chances of CKD over a four-year follow-up in general population (Table 3). After adjustment for sociodemographic variables, this pattern remained, but the magnitude of the associations decreased substantially. In the final model, it was possible to observe that only the 4^th^ quartile remained statistically significant, showing that men with PWV higher than 10.3 m/s and women with PWV higher than >9.6 m/s, presented 42% (95% CI: 1.05;1.92) more chances of CKD after four years of follow-up. Analysis of normotensive, non-diabetic participants yielded equivalent results, but with greater magnitudes of associations (Table 4). In the final model including only normotensive, non-diabetic participants, there was a clear dose-response gradient in the association between cfPWV quartile and the chances of CKD, reaching an OR of 1.81 (95%CI: 1.14;2.86) among individuals in the upper quartile in comparison with those in the lower one (Table 4).

**Table 3:** – Association between quartiles of pulse wave velocity and the incidence of chronic kidney disease in the entire sample, after four years of follow-up (2008/2010–2012/2014)

Models	Chronic Kidney Disease (N:11,647) OR (CI 95%)
Univariate model	
1^st^ quartile	Ref
2^nd^ quartile	1.74 (1.31;2,31)***
3^rd^ quartile	2.16 (1.64;2.83)***
4^th^ quartile	4.14 (3.22;5.33)***
Model 1: model 0 + age, sex, race/color, and education	
1^st^ quartile	Ref
2^nd^ quartile	1.46 (1.09;1.94)*
3^rd^ quartile	1.50 (1.13;1.99)**
4^th^ quartile	1.94 (1.47;2.55)***
Model 2: model 1 + smoking, physical activity, total cholesterol/HDL-C and BMI	
1^st^ quartile	Ref
2^nd^ quartile	1.39 (1.04;1.86)*
3^rd^ quartile	1.42 (1.06;1.88)*
4^th^ quartile	1.79 (1.36; 2.37)***
Final model: model 3 + DM, Use of antihypertensive, SBP, HR and CVD	
1^st^ quartile	Ref
2^nd^ quartile	1.32 (0.98;1.78)
3^rd^ quartile	1.31 (0.98; 1.76)
4^th^ quartile	1.42 (1.05;1.92)*

OR: odds ratio obtained by multiple logistic regression. CI: confidence interval. BMI: Body mass index. HR: heart rate. SBP: systolic blood pressure. CVD: cardiovascular disease. *p<0,05 **p<0,01 ***p<0,001

**Table 4:** Association between quartiles of pulse wave velocity and the incidence of chronic kidney disease in the subsample of normotensive, non-diabetic participants, after four years of follow-up (2008/2010–2012/2014)

*Models*	*Chronic Kidney Disease (N:7,189) OR (CI 95%)*
Univariate model	
1^st^ quartile	Ref
2^nd^ quartile	1.99 (1.35;2.94)***
3^rd^ quartile	2.39 (1.62;3.62)***
4^th^ quartile	3.49 (2.35;5.20)***
Model 1: model 0 + age, sex, race/color, and education	
1^st^ quartile	Ref
2^nd^ quartile	1.66 (1.12;2.46)*
3^rd^ quartile	1.65 (1.17;2.47)*
4^th^ quartile	1.79 (1.17;2.74)**
Model 2: model 1 + smoking, physical activity, total cholesterol/HDL-C and BMI	
1^st^ quartile	Ref
2^nd^ quartile	1.58 (1.06;2.35)*
3^rd^ quartile	1.61 (1.07;2.41)*
4^th^ quartile	1.76 (1.14;2.70)**
Final model: model 3 + SBP, HR and CVD	
1^st^ quartile	Ref
2^nd^ quartile	1.61 (1.08;2.41)*
3^rd^ quartile	1.63 (1.07;2.47)*
4^th^ quartile	1.81 (1.14;2.86)*

OR: odds ratio obtained by multiple logistic regression. CI: confidence interval. BMI: Body mass index. HR: heart rate. SBP: systolic blood pressure. CVD: cardiovascular disease *p<0,05 **p<0,01 ***p<0,001

## Discussion

In this large Brazilian multicenter cohort of adults, the chances of developing CKD in four years of follow-up, based on eGFR and/or ACR, was 42% higher in individuals in the upper quartile relative to those in the lower one, after adjustments for sociodemographic, behavioral, and clinical characteristics. When only baseline normotensive, non-diabetic individuals were accounted for, the magnitudes of the associations of cfPWV quartiles and CKD, compared to the lower quartile, were greater than those observed in the overall sample, and progressive - almost doubling in the upper quartile.

Studies suggest that cfPWV>10 m/s increases cardiovascular risk, being a major marker of clinical risk.^8,28^ In this study, the 4^th^ cfPWV quartile corresponds to values of cfPWV above 10.3 m/s in men and above > 9.6 m/s in women. A global meta-analysis of 167 studies, totaling 509,743 subjects, provided significant information on sex differences in cfPWV measurements, in which men had greater arterial stiffness than women, mainly during young-adult age and up to 60 years old, justifying the use of cfPWV data divided into sex-specific quartiles.^29^

The findings of this study corroborate other longitudinal studies that investigated the relations between arterial stiffness and incidence of renal dysfunction defined according to eGFR in the general population^24,26,11^ and in individuals with CKD^7,10^ or comorbidities, such as DM^30^and hypertension.^12^ Itano et al. (2020) also found individuals in the upper arterial stiffness quartile had increased CKD incidence over a mean follow-up of 3.1 years when compared with the other quartiles grouped as a reference. However, unlike this study, they used a cardio-ankle vascular index and the highest quartile corresponded to >8.1 m/s.^26^ Townsend (2018) followed up 2,795 participants, mean age of 60 years, for 4.9±2.1 years and found that individuals in the upper cfPWV tercile (>10.3 m/s) had 37% more risk of developing CKD (95%CI: 1.05-1.80), as well as 25% greater risk of having end-stage renal disease or having their eGFR reduced by half (HR: 1.25; 0.98-1.58).^7^

The results of this study on the whole cohort make significant contributions to the few longitudinal population-based studies that used ACR as a marker of kidney function. Findings from a Chinese cohort^31^ of 7,154 individuals, with a mean age of 54, showed a linear association between arterial stiffness and risk of CKD, in which every 1 m/s increase in PWV was associated with a 15% higher chance of proteinuria (95%CI: 1.07-1.23) after a three-year follow-up. However, smaller cohort studies failed to reveal significant associations between cfPWV values and the incidence of microalbuminuria in models fully adjusted for cardiovascular risk factors. In the Framingham Offspring study, higher cfPWV was modestly associated with microalbuminuria in 568 participants with ACR <30 mg/g at baseline, following adjustment for age and sex, but not in the final model adjusted for all risk factors after a 7- to 10-year follow-up.^15^ Significant associations between cfPWV and ACR are more common in cross-sectional studies.^32^

In normotensive, non-diabetic individuals, the magnitude of these associations was slightly higher, suggesting that the impact of higher cfPWV on CKD risk is more pronounced in previously healthy individuals. This finding may reflect that: 1) higher arterial stiffness is per se associated with the incidence of CKD and not a consequence of hypertension and diabetes, or 2) residual confounding of hypertension and diabetes in the models applied to the general population. Significant relations between cfPWV and kidney function have been demonstrated in normotensive individuals with mild to moderate renal failure.^33^ In a cross-sectional analysis, arterial stiffness was associated with higher odds of CKD and renal dysfunction in individuals without hypertension and diabetes, suggesting that the relations between arterial stiffness and kidney dysfunction are not entirely explained by these conditions.^34^ Considering that greater arterial stiffness increases the risk of hypertension and diabetes,^23,24^ it is possible to hypothesize that it could also be directly implicated in the genesis of CKD, regardless of hypertension and diabetes. Thus, it suggests that the effect of increased arterial stiffness on the incidence of CKD is independent of hypertension and/or diabetes, the major risk factors for renal dysfunction.^12^ The lower magnitude of the association in the general population can be justified by a possible residual confounding by these factors.

Some mechanisms may explain associations between increased arterial stiffness and higher incidence of CKD. Owing to its low vascular impedance, renal circulation is sensitive to blood pressure oscillations and increased pulsatility, which results from increased arterial stiffness.^35^ Greater stiffness of the media layer in large arteries may affect the ability of renal vessels to attenuate blood pressure changes with each systolic ejection. Hence, as the aorta becomes stiffer, the pulsatile stress in peripheral blood vessels increases, leading to microvascular damage, hyperfiltration, and glomerular hypertrophy and sclerosis, which result in decreased filtration surface area and lower GFR.^35,36^ In turn, hemodynamic stress in renal vessels may lead to endothelial dysfunction and microvascular ischemia, which interfere with the permeability of the glomerular barrier^12,31^ and allow greater urinary excretion of albumin.^37^ These mechanisms may occur even in individuals free of hypertension and diabetes.

The strengths of this study include the large cohort size, comprehensive and rigorous data collection and high retention rate (92.7%), ascertainment of CKD according to TFG or ACR, and analysis of a subset of normotensive, non-diabetic individuals. However, the relationship between arterial stiffness and CKD may be more fully examined with a longer follow-up, given the slow progression of renal dysfunction,^38^ particularly albuminuria. Also, eGFR and ACR were estimated at a single time point. Although this is a widespread practice in large epidemiological studies such as ELSA-Brasil, the definition of CKD according to renal changes persisting for three months or more^38^ was not considered. Intra-individual variability in urinary albumin and creatinine excretion has also been reported.^39^

## Conclusion

Higher arterial stiffness increases the risk of CKD in the general population and in individuals free of hypertension or diabetes, suggesting that the association found is not dependent on these comorbidities. Considering that CKD increases the risk of death, cardiovascular events, and morbidity, these results emphasize the importance of vascular health to prevent CKD development.
